# Oncolytic herpes simplex virus tumor targeting and neutralization escape by engineering viral envelope glycoproteins

**DOI:** 10.1080/10717544.2018.1534895

**Published:** 2018-12-04

**Authors:** Xiao-Qin Liu, Hong-Yi Xin, Yan-Ning Lyu, Zhao-Wu Ma, Xiao-Chun Peng, Ying Xiang, Ying-Ying Wang, Zi-Jun Wu, Jun-Ting Cheng, Jia-Fu Ji, Ji-Xin Zhong, Bo-Xu Ren, Xian-Wang Wang, Hong-Wu Xin

**Affiliations:** a Faculty of Medicine, The Second School of Clinical Medicine, Yangtze University, Nanhuan, Jingzhou, Hubei, China;; b Laboratory of Oncology, Faculty of Medicine, Center for Molecular Medicine, School of Basic Medicine, Yangtze University, Jingzhou, Hubei, China;; c Faculty of Medicine, Department of Biochemistry and Molecular Biology, School of Basic Medicine, Yangtze University, Jingzhou, Hubei, China;; d Department of Nursing and Medical Imaging Technology, Yangtze University, Jingzhou, Hubei, China;; e Star Array Pte Ltd, JTC Medtech Hub, Singapore, Singapore;; f Institute for Infectious Diseases and Endemic Diseases Prevention and Control, Beijing Center for Diseases Prevention and Control, Beijing, China;; g Faculty of Medicine, Department of Pathophysiology, School of Basic Medicine, Yangtze University, Jingzhou, Hubei, China;; h Key Laboratory of Carcinogenesis and Translational Research (Ministry of Education), Department of Gastrointestinal Surgery, Peking University Cancer Hospital and Institute, Haidian, Beijing, China;; i Cardiovascular Research Institute, Case Western Reserve University, Cleveland, OH, USA;; j Faculty of Medicine, Department of Laboratory Medicine, School of Basic Medicine, Yangtze University, Jingzhou, Hubei, China

**Keywords:** Oncolytic virotherapy, herpes simplex virus, envelope glycoprotein, tumor targeting, immune escape, neutralization antibody

## Abstract

Oncolytic herpes simplex viruses (oHSVs) have been approved for clinical usage and become more and more popular for tumor virotherapy. However, there are still many issues for the oHSVs used in clinics and clinical trials. The main issues are the limited anti-tumor effects, intratumor injection, and some side effects. To overcome such challenges, here we review the genetic engineering of the envelope glycoproteins for oHSVs to target tumors specifically, and at the same time we summarize the many neutralization antibodies against the envelope glycoproteins and align the neutralization epitopes with functional domains of the respective glycoproteins for future identification of new functions of the glycoproteins and future engineering of the epitopes to escape from host neutralization.

## 1. Introduction

Nowadays, cancer is still a big problem for a human. In the past several decades, the main methods of treatment for cancer include surgery, chemotherapy, radiotherapy, targeted therapy, immunotherapy, and so on. Although these therapies prolonged the median survival time for patients, they have severe side effects and other shortcomings. Their unsatisfactory therapeutic effects are partly caused by complex genetic and epigenetic changes (Guo et al., 2013, 2016; Hu et al., 2017; Guo et al., 2018), drug-resistant tumor stem cells (Hari, 2011; Xin, 2012, 2013a, 2013b, 2016), and inhibitory tumor microenvironments (Liu et al., 2017). Therefore, it is very important to explore new methods to treat cancer.

According to the recent reports, great progress have been made on oncolytic herpes simplex viruses (oHSVs), a new method to detect and treat tumors (Zhang et al., 2016; Wang et al., 2018; Wu et al., 2018). Oncolytic viruses (OVs) are a kind of viruses that can infect and replicate in cancer cells but spare the normal cells (Russell et al., 2012). In 2015, talimogene laherparepvec (T-VEC) had been approved by the US Food and Drug Administration (FDA) to treat melanoma (Pol et al., 2016; Rehman et al., 2016). G207, a multimutated oHSV, has been shown to be safe and effective to treat pediatric supratentorial tumors in phase I clinical trial (Waters et al., 2017). The most common side effect of it is the acute, transient flu-like symptom (Senzer et al., 2009). All of these outstanding achievements have demonstrated the promising anti-tumor prospect of oHSVs.

These successes and unresolved issues encouraged us and others to explore new ideas to make better oHSVs. One big issue is that most oHSVs infect not only cancer cells but also normal cells, leading to inefficiency and side effects, such as fatigue, nausea, influenza-like illness, vomiting, and headache (Pol et al., 2016; Rehman et al., 2016; Fountzilas et al., 2017). In addition, engineered oHSVs were often attenuated so that they have less pathogenicity in human, but the ability of replication and oncolysis is also attenuated. Many of the engineered oHSVs are to delete or inactivate viral genes that are essential in pathogenicity, for instance, infected cell polypeptide (ICP) 34.5 and ICP6 (Chou et al., 1990; MacLean et al., 1991; Yazaki et al., 1995; Todo et al., 2001; Hu et al., 2006; Kemeny et al., 2006). The ability of these attenuated oHSVs to replicate in host cells was wakened at least partly and the efficacy of killing tumor cells was affected (Markert et al., 2000). According to the reports, some cancer cells are resistant to oHSVs because of a lack of the natural receptor nectin-1 (Huang et al., 2007; Yu et al., 2007). Deletion of the neural pathogenicity determinant thymidine kinase in engineered oHSVs leads to that the acyclovir or ganciclovir cannot control the possible side effects caused by it (Martuza et al., 1991). In consideration of these unresolved issues, the ideal oHSVs should infect only the cancer cells while keeping their ability to replicate and destroy cancer cells. Hence, it is very necessary to engineer oHSVs envelope glycoproteins that target the receptors overexpressed in cancer cells, increasing their antitumor efficiency and reducing side effects.

Another big issue is that the most way to treat cancers with oHSVs is intratumoral injection and this limits the use of oHSVs. The systemic injection of oHSVs may become inefficient partly due to preexisting neutralization antibodies against anti-HSV envelope glycoproteins in most patents and evoke systematic antivirus immune responses against oHSVs (Todo et al., 2001; Hellums et al., 2005; Varghese et al., 2006; Farrell et al., 2008). If oHSVs can get to tumor sites through the circulatory system and avoid the attack of the immune system by engineering the envelope glycoprotein neutralization epitopes, the use of oHSVs to treat tumor will be more effective and convenient.

Here we review the genetic engineering of the functional domains of the envelope glycoproteins and at the same time we summarize the many neutralization antibodies against the envelope glycoproteins and align the neutralization epitopes with functional domains of the respective glycoproteins for future studies.

## 2. Tumor targeting of oHSVs by engineering their envelope glycoproteins

**Figure 1.**
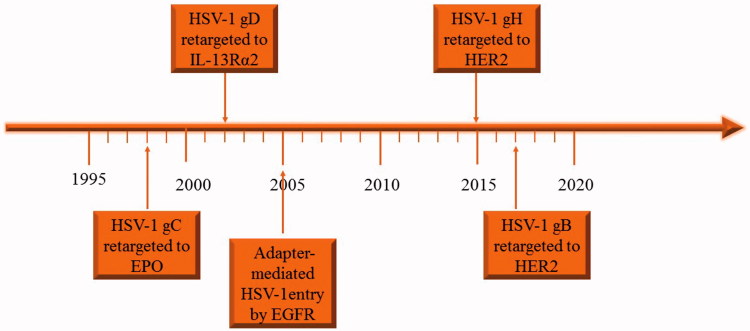
The timeline of engineering the glycoproteins of HSV to retarget to tumor cells.

Genetic engineering of envelope glycoproteins makes oHSVs to target tumors specifically. Engineering envelops glycoproteins of oHSVs began with the engineering of the glycoprotein C (gC) by Laquerre et al. (1998) (Figure 1). Zhou et al. first engineered gD in 2002 and Gatta et al. first engineered gH in 2015 and only recently Petrovic et al. first engineered gB in 2017, respectively (Figure 1) (Zhou et al., 2002; Gatta et al., 2015; Petrovic et al., 2017). Nakano et al. first used soluble molecule bridge (adaptor) to redirect the HSV-1 to the epidermal growth factor receptor (EGFR) (Nakano et al., 2005). Single-chain variable fragment (scFv) of antibodies may have high affinity to antigens (e.g. EGFR) overexpressed in cancer cells and have been used to engineer oHSV envelope glycoproteins to target tumor (Jiang et al., 1998; Kuan et al., 2000). Another approach is to insert the natural ligand of special receptor that is enriched in cancer cells to the surface of the virus, for example interleukin-13 receptor α2 chain (IL-13Rα2) and N-terminal fragment of urokinase-type plasminogen activator are in enriched in some kind of cancer cells (Debinski et al., 1999; Zhou et al., 2002; Kamiyama et al., 2006).

### 2.1. Tropism retargeting of oHSVs based on a modification of gC

**Figure 2.**
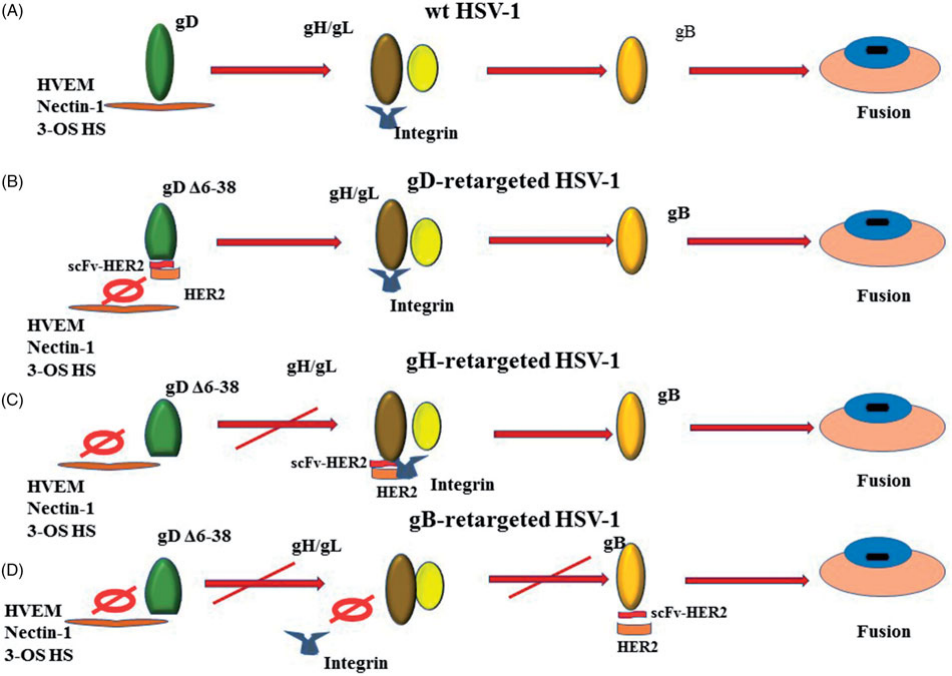
Four different ways for HSVs to enter into host cells. (A) A wild type HSV enters into a host cell; (B) an oHSV retargeted to Her2-scFv-enginnered gD; (C) an oHSV retargeted to Her2-scFv-enginnered gH; (D) an oHSV retargeted to Her2-scFv-enginnered gB.

In the process of the virus entering into a host cell, the first step is the attachment of a virus to a cell. In HSVs gC is the major glycoprotein that can bind to the heparan sulfate proteoglycans (HSPGs) and induce attachment, and the binding of gC with glycosaminoglycan can induce HSV attachment to the cell surface (Herold et al., 1991). gD may bind with one of its receptors without gC (Montgomery et al., 1996; Cocchi et al., 1998; Geraghty, 1998; Shukla et al., 1999). The gB binding to proteoglycans can induce the binding of the virus to cells in the absence of gC and may precede gD function (Herold et al., 1994; Krummenacher et al., 2005). The interaction of gB or gC with cell-surface heparan sulfate (HS) on the cell surface can facilitate the binding of gD with its receptors (Figure 2(A)) (Tiwari et al., 2007).

According to the reports below, gC can be engineered to retarget the special receptors on cell surface. Laquerre et al. first retargeted the HSV-1 to EPO receptor, in which the gC of HSV-1 was engineered, and the HS-binding region was replaced with erythropoietin hormone (EPO) ligand, which altered the entry of virus into cells, such that it was endocytosed by a pathway that did not lead to a productive infection (Laquerre et al., 1998). This study opened the new door for oHSVs to retarget the non-HSV receptors. In a similar method, the gC of HSV-1 was engineered to express a pre-S1 active peptide that binds with its hepatitis B virus receptor expressed on liver cells (Argnani et al., 2004). Grandi et al. replaced the HS-binding domain of gC with His-tag sequence so as to be retargeted to the 293 6 H cells expressing a cell surface pseudo-His-tag receptor (Grandi et al., 2004). The glycoprotein gC in HSV-1 has been engineered to target the special receptor, NMDA receptor NR1 subunit, so that the virus can specifically infect the cells containing NR1 (Cao et al., 2010). Zhou et al. reported that HSV-1 was retargeted to a specific receptor IL13Rα2 expressed in malignant glioma, by ablating the HS-binding sites of gB and gC, and the insertion of IL-13 in the N-terminal of gC and gD, but the recombinant virus can still infect normal cells by interaction with HVEM or nectin 1 (Zhou et al., 2002). The glycoprotein gC can also be retargeted to the human glioma cells through the ligand human glioma-specific peptide sequence (denoted as MG11) (Ho et al., 2010).

In addition, a specific mutant EGFR, EGFRvIII, overexpressed on breast carcinomas, lung carcinomas, and other tumors, can be recognized by scFv mutant receptor 1 (MR1) (Wikstrand et al., 1995; Lorimer et al., 1996). MR1-1, derived from MR1 scFv, has increased affinity to EGFRvIII than MR1 (Kuan et al., 2000). HS binding domain can be deleted and replaced by the scFv MR1-1, and the MR1-1-modified gC had 5-fold increased infectivity for EGFRvIII positive cells. The authors stated that retargeting the virus should enhance the tumor targeting specificity, tumor-killing ability, and safety (Grandi et al., 2010).

However, retargeting of gC may not be useful for tumor targeting as the virus can still attach to the normal receptors. The gC retargeting described above was done to use a novel receptor for virus attachment but this does not prevent virus entry through the widely distributed cognate gD receptors HVEM and nectin-1. Blocking HS binding reduces virus infectivity by at least 10-fold, which may be reversed by gC retargeting. Thus, gC retargeting favors infection of target cells but does not prevent off-target infection.

### 2.2. Tropism retargeting of oHSVs based on a modification of gD

HSV-1 entry into target cells requires gD, gB, and heterodimer gH/gL, as well as one of the three gD receptors (Herold et al., 1991) (Figure 1(A)). These four glycoproteins are sufficient to infect host cells even though the other glycoproteins of HSV-1 are absent (Turner et al., 1998). The gD of HSV-1 can interact with three different cellular receptors, namely nectin-1, herpesvirus entry mediator (HVEM) and 3-O-sulfated heparan sulfate (3-OS HS) (Montgomery et al., 1996; Cocchi et al., 1998; Geraghty, 1998; Shukla et al., 1999). HSV cannot bind with both HVEM and nectin-1 simultaneously. When HSV interacts with nectin-1 directly, the soluble nectin-1 can block binding of the virus with HVEM, and vice versa (Geraghty, 1998). The binding of glycoprotein gD with one of its receptors triggers the ability of gB to cause membrane fusion, and the gD determines the tropism of the HSV to the host cells. In the process of infection, the N-terminus of gD binds to the HVEM or nectin-1 and the C-terminus is opened from binding to N-terminus (Fusco et al., 2005). Then the afresh exposed section of gD will interact with gH/gL to induce the structural change of gH/gL, especially at the N-terminus of gH and the C-terminus of gL, so as to activate them (Atanasiu et al., 2010b; 2013). The activated gH/gL, in turn, activates the gB to mediate the membrane fusion (Atanasiu et al., 2010a, 2010b). In addition, gE and gI are very important for HSV to spread intercellularly, but are dispensable for its entry into host cells (Johnson & Huber, 2002). The gC-null virus can still enter into the cells, whereas its virulence was severely attenuated (Drolet et al., 2004; Nicola & Straus, 2004). Although gC is not necessary for HSV-1 to enter into host cells, the binding of it to the complement C3b can inhibit complement activation so as to protect the virus from antibody neutralization (Friedman, 2003).

One strategy to alter the tropism is to engineer the gD of HSV so that HSV can be retargeted to the receptors that are expressed specifically or preferentially in cancer cells but not in normal cells (Figure 2(B)). In this way, oHSVs can be retargeted to the cancer cells and detargeted from the normal cells (Zhou & Roizman, 2006; Menotti et al., 2008). At the same time, the virus can retain the full ability of replication and oncolysis. According to the report, the N terminus is necessary for the binding of HSV to 3-OH-SH and HVEM, but not necessary for nectin-1 (Yoon et al., 2003). Hence, the mutation the N terminus can ablate the interaction of HSV to HVEM and 3-OH-SH at the same time, but not nctin-1.

It has been reported that N-terminus of gD can tolerate long insertions, whereas AA (AA) residues next to the gD Ig-like V-type core tolerated no more than 60 AAs (AAs) insertions (Fan et al., 2017). The AA 61-218 of gD is not necessary for the virus entry cells and encode executable functions, and the structure of the residual portion of gD cannot be changed during insertion ligand in gD (Zhou & Roizman, 2007). IL-13 was inserted into gD of HSV so that the virus can be retargeted to the cells expressing IL-13 receptor α2 (Zhou & Roizman, 2006).

However, full retargeting can greatly compromise virus infectivity due to improper processing of the retargeted molecules (Zhou & Roizman, 2006). Simply put, less retargeted molecules (e.g. retargeted gD) are present in the virus particle reducing infectivity (Petrovic et al., 2017). This occurred in the IL-13 receptor α2 retargeting that could not detect retargeted gD although levels of other viral proteins were normal (Zhou & Roizman, 2006). This can be overcome to some degree by creating mutations in the viral fusion functions that enhance virus entry. Furthermore, to avoid the nonspecific infection and side effects complete and irreversible detargeting of HSV from normal cells is very important. HSV-1 retargeted to epithelial cell adhesion molecule (EpCAM) have also been reported and the intercellular spread of the retargeted oHSV depends on the expression of EpCAM (Shibata et al., 2016). Retargeting HSV-1 to urokinase plasminogen activator receptor (uPAR) and the GD2 have been explored (Zhou & Roizman, 2007; Fan et al., 2017).

Human epidermal growth factor receptor 2 (HER-2) is a member of epidermal growth factor receptor (EGFR) family, which is overexpressed in breast cancer, ovary cancer, uterine endometrioid carcinoma, gastric carcinomas, glioblastomas, etc (Jackson et al., 2013). The overexpression of HER2 usually represents that the tumor is more progressive and has a poorer prognosis (Barros et al., 2010). HSV R-LM113 retargeted to HER-2 is engineered by deletion of the AAs 6-38 in gD and replacement of it with scFv to HER-2 so as to bind HER-2 but not to bind HVEM and nectin-1 (Figure 2(B)) (Menotti et al., 2008). In the HER-2 retargeted HSV R-LM249, whose AAs 61-218 of gD was replaced with Ig-folded scFv to HER-2, AAs 61-218 is critical to gD-nectin-1 interaction and the deletion of it prevent from any possibility to revert into WT type HSV (Menotti et al., 2009). R-LM249 reserves the thymidine kinase gene to guarantee the efficacy of acyclovir on the oHSV in a worst-case scenario (Reisoli et al., 2012; Nanni et al., 2013). The preclinical studies showed that R-LM113 played a role in cancer treatment and prolonged survival in immunodeficient and immunocompetent mice, whereas R-LM249 can infect and kill solely HER-2-overexpressed cancer cells and can reduce the tumor growth and inhibit carcinomatosis efficiently (Nanni et al., 2013).

On the study of retargeting oHSVs to EGFR and CEA, which is overexpressed in cancer cells, the AAs 2-24 of gD was deleted and a single AA substitution, Y38C, was introduced to ablate the responsiveness to nectin-1. In addition, the efficiency of infection of human glioblastoma multiforme (GBM) with the oHSV was further improved by the simultaneous introduction of mutations in gB (D285N/A549T), a pair of fusion-accelerating mutations and life was prolonged up to 75% more in the mice model (Uchida et al., 2013).

### 2.3. Tropism retargeting of oHSVs based on a modification of gH

In the process of virus entry into host cells, gH/gL plays a role to transmit signal from gD to gB, and then gB is activated to trigger virus-cell fusion (Figure 2(A)) (Atanasiu et al., 2010a). During signal transduction, integrins αvβ6 or αvβ8 can serve as receptors of gH/gL to mediate virus entry into cells by activation of gH and dissociation of gL from gH (Gianni et al., 2013). To complete such dissociation, the activation of gD by binding to one of its receptors, nectin1 or HVEM, is required (Gianni et al., 2015).

Gatta et al. first reported that the chimeric gH redirected HSV, R-VG809, was engineered to retarget to the cancer cells by inserting the scFv-HER2 between AA 22 and 23 of gH and delete the AAs 6-38 of gD (Figure 2(C)). The recombinant virus R-VG809 has as good as, if not better, the ability of replication and killing cancer cells than those gD retargeted viruses like R-LM113 and R-LM249. Growth efficiency of R-VG809, R-LM113, and R-LM249 in cancer cells are as good as wt HSV R-LM5 (Gatta et al., 2015). Some literature demonstrated that the deletion of 28 residues at the N terminus of glycoprotein gH (gHΔ48/gL) of HSV-2 can induce low-level fusion of HSV-2 to cells in the absence of gD and/or its receptor (Atanasiu et al., 2013). Hence, the authors proved that the gH/gL engineered to retarget tumor cells may have a promising future.

### 2.4. Tropism retargeting of oHSV based on modification of gB

The glycoprotein B (gB) as a viral fusogen performs fusion by the hydrophobic and hydrophilic residues of its fusion domain to associates with lipid membranes (Hannah et al., 2009). Normally, a conserved heterodimer gH/gL is required for gB to perform fusion in addition to other unconserved glycoproteins (Cooper & Heldwein, 2015). The glycoprotein gB can be activated by release of its cytodomain under the action of a ‘wedge’, N-terminal of gH cytotail and the fusion levels are proportionate to the length of gH cytotail (Rogalin & Heldwein, 2015).Recently, the recombinant HSV, R909 was engineered to retarget tumors and detarget from its natural gD receptors by insertion of HER2 scFv between AAs 43 and 44 of gB and deletion of the AAs 6-38 of gD (Figure 2(D)) (Petrovic et al., 2017). HER2 expressed at cell membrane directly activate the fusogenic domain of the chimeric gB once the scFv binds to HER2. The results showed that the gB-retargeted oHSV R-909 has a very similar virus growth, plaque size, and killing ability with the virus that is retargeted through gH.

The retargeting efficiency of oHSVs is likely to be determined by position and type of ligands of the receptors, the number of receptors in host cells and the affinity of ligands to receptors (Uchida et al., 2013). The functions of all the oHSVs engineered by glycoproteins (including R-LM113, R-LM249, R-809, and R-909) engineered and tested by Petrovic et al. have been proved impaired compared with the wild types (Petrovic et al., 2017). At the same time, the hyperactive gB allele D285N/A549T can increase the yield of the gD retargeted virus in the host cells, so the hypersensitization of gB can complement the impaired gD function (Uchida et al., 2010). The glycoprotein gB mutants A855V and A874P can mediate low-level membrane fusion in the absence of gD or gH/gL (Silverman et al., 2012). According to the report, the combination of gH (KV) and gB (S688N) enabled the virus to enter the host cells as efficiently as the gB hyperactive mutations D285N/A549T (gB:NT) in the absence of natural receptors, and the mutants of gB can enhance entry of viruses to host cells, whereas the mutants of gH can enhance the secondary virus spread between cells (Uchida et al., 2013). This indicates that the gB mutant can induce membrane fusion through the way that is still not clear.

In summary, oHSVs have been made by engineering their envelope glycoproteins gC, gD, gH, or gB retargeted to tumors with scFv/ligand, and detargeted from their natural receptors, nectin-1 or HVEM (Table 1).

**Table 1.** oHSVs engineered at their envelope glycoproteins.

Virus	oHSV	Modified glycoprotein	Additional modification	Retargeted to	Detargeted from	References
HSV-1	KgBpK2gC- EPO2	gC	gC_Δ1–161 _gB: HS binding site deletion	EPO	The gC and gB receptor HS	Laquerre et al., 1998
HSV-1	KgBpK^-^gC: preS1ap	gC	preS1ap: gC_Δ149–442 _preS1: gC_Δ149–213 _gB: lysine-rich domain deletion	preS1 peptide	The gB and gC receptor: HS	Argnani et al., 2004
HSV-1	gCmutHis-tag	gC	gC_Δ33–174_	His-tag	gC receptor HS	Grandi et al., 2004
HSV-1	gC-ZZ protein	gC	gC: Staphylococcus A protein ZZ domain replaced bdnf domain.	NMDA receptor NR1 subunit	gC receptor HS	Cao et al., 2010
HSV-1	R5111	gC	gC_Δ136–152_: IL-13 replaced AA148 gB_Δ68–77 _gD: IL-13 insertion after AA24	IL13Rα2 receptor	gB and gC receptor: HS	Zhou et al., 2002
HSV-1	MG11-pCONGA	gC	gC_Δ33–123_	Human glioma cells	gC receptor HS	Ho et al., 2010
HSV-1	MR1-1/EGFRvIII	gC	gC_Δ33–174_	EGFRvIII	gC receptor HS	Grandi et al., 2010
HSV-1	R-LM113	gD	gD_Δ6–38_	HER2	nectin1 and HVEM	Menotti et al., 2008
HSV-1	R5141	gD	gC_Δ1–132 _Poly(K) deletion in gB gD_Δ1–32_	IL-13Rα2	HS HVEM Nectin-1	Zhou & Roizman, 2006
HSV-1	KGNEp	gD	gD_Δ2–24 _A hyperactive allele, D285N/A549T (gB:NT).	Epithelial cell adhesion molecule (EpCAM)	Nectin-1 and HVEM	Shibata et al., 2016
HSV-1	R5322	gD	gD_Δ1–32 _mutations at 34, 38, 215, 222, and 223 in gD, 62–218 deletion	Urokinase plasminogen activator (uPA)	Nectin-1 and HVEM	Zhou & Roizman, 2007
HSV-1	R-LM249	gD	gD_Δ61–218_	HER2	Nectin-1 and HVEM	Menotti et al., 2009
HSV-1	KNE (retargeted to EGFR) and KNC (retargeted to CEA)	gD	gD_Δ2–24 _Y38CgB: D285N/A549T	EGFR CEA	Nectin-1 and HVEM	Uchida et al., 2013
HSV-1	R-809	gH	gD_Δ6–38_	HER2	Nectin-1 and HVEM	Gatta et al., 2015
HSV-1	R-909	gB	gD_Δ6–38_	HER2	Nectin-1 and HVEM	Petrovic et al., 2017

The choice of receptors for targeting is also a complex issue. With few exceptions, tumors express receptors that are shared by normal tissues. To increase the specificity, target mutated neoantigens would be a good choice. Targeting cancer stem cells by their cell surface markers, such as CD44, CD133, and EpCam would avoid nonspecific targeting to differentiated normal cells. Most cancers are highly heterogeneous making targeting difficult. Targeting cancer stem cells would also increase the chance of targeting heterogeneous differentiated cancer cells.

In addition, the cultivation of the retargeted oHSVs requires healthy cells expressing the retargeted receptors/ligands to produce clinical-grade oHSVs. Although the retargeted oHSVs can be cultivated in cancer cells that overexpress the targeted receptors/ligands but may not be well cultivated in healthy cells.

To achieve this aim, the insertion of a 22-AA peptide (named GCN4 derived from a yeast transcription factor) in gH of R-LM113 produced a new recombinant virus named R-213, which was retargeted to HER-2 through the insertion of HER-2 scFv in gD. An artificial receptor GCN4R is expressed by Vero cells, whose N-terminus consists of a scFv to GCN4, and it can interact with the GCN4 present in gH of R-213. The results showed that R-213 replicates in GCN4R expressing Vero cells as well as R-LM113 in SK-OV-3 cells (Leoni et al., 2017). Using gD to retarget the virus to HER2 and gB to retarget to GCN4R were also tested successfully (Petrovic et al., 2018). Insertion of two ligands HER2 and GCN4R in gD at the same time can also be used to achieve this aim (Leoni et al., 2018).gD can also be retargeted by bispecific adapters, which do not need the engineering of the gD to retarget the specific receptors (Waehler et al., 2007). This method is based partially on the report that the HSV infection through HVEM/nectin-1 can be blocked by soluble type of these receptors (Montgomery et al., 1996; Whitbeck et al., 1997; Geraghty, 1998; Krummenacher et al., 1999; Lopez et al., 2001). Hence, the adaptor composed of the gD binding region of any one receptor and special ligand or scFv can make the virus retarget to the special receptor and detarget from its natural receptors. Nakano et al. first made the adaptor protein P-V528LH, which is composed of the gD-binding variable domain of nectin-1 fused to a single-chain antibody (528LH) recognizing the EGF receptor. It can induce the HSV-1 to enter the cells through a new receptor, but the infection by nectin-1 was not blocked (Nakano et al., 2005). Baek et al. used a bispecific adaptor with a CEA-specific single-chain antibody fused to gD binding region of HVEM to retarget cancer cells that over-express CEA and avoid the binding to the HVEM (Baek et al., 2011).

According to the reports, at least 30 miRNAs have been found to be deferentially expressed between normal cells and glioblastoma, neurons or neural progenitor cells (Riddick & Fine, 2011; Karsy et al., 2012). The difference can be used to retarget HSV-1 to tumor cells. The recognition sequence for the miR-124 was engineered into the 3′UTR of the essential gene ICP4 of an EGFR/EGFRvIII-specific HSV to prevent virus replication in normal cells, which can improve its safety (Mazzacurati et al., 2015).

## 3. Function analysis and neutralization escape of oHSV envelope glycoproteins

**Figure 3.**
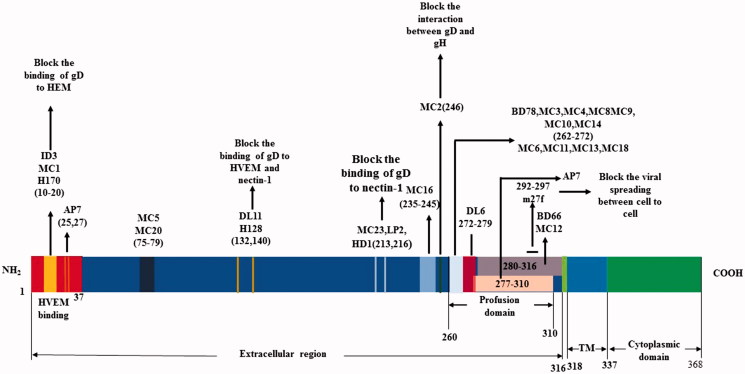
Alignment of neutralization epitopes with functional domains of gD.

**Figure 4.**
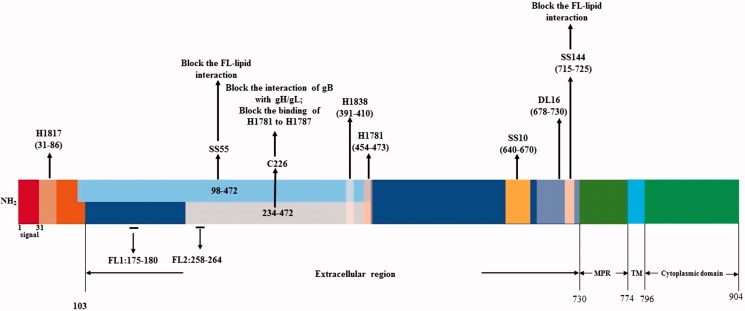
Alignment of neutralization epitopes with functional domains of gB.

oHSVs have a great potential to target tumors but produce a large number of neutralization antibodies against their envelope glycoproteins (Peng et al., 1998; Whitbeck et al., 1999; Cairns et al., 2006; Bender et al., 2007; Eisenberg et al., 2012), which limits their oncolytic effects and systemic application (Fu & Zhang, 2002; Varghese et al., 2007; Kulu et al., 2009; Coffin, 2015). Neutralization epitopes of envelope glycoproteins can be used to identify new function of glycoproteins and engineered for oHSVs to escape from host neutralization. To promote these studies, we aligned the neutralization epitopes with the functional domains of oHSV envelope glycoproteins (Figures 3 and 4).

The neutralization antibodies react mainly against gD or gD + gB and are type specific (Cairns et al., 2015). The gC and gG of HSV-1 can generate equivalent antibodies in blood samples for the determination of HSV-1 and HSV-2 serotypes (Scheper et al., 2010). Neutralization antibody responses can be stimulated in animal models (Eing et al., 1989; Peng et al., 1998; Awasthi et al., 2011; 2014). It has been reported that gC of HSV can generate neutralizing antibodies in animal models (Adamiak et al., 2010; Awasthi et al., 2011). It is also reported that the gC and gE can reduce neutralization antibody and complement responses (Friedman, 2003). Many studies have explored the monoclonal antibody (McAb or MAb) epitopes in the glycoproteins and how they neutralize the virus (Figures 3 and 4). MAbs are a kind of useful tool to study the structure and function of glycoproteins.

### 3.1.Alignment of neutralization epitopes with functional domains of gD

It has been known that gD can induce potent neutralizing antibodies (Para et al., 1985). gD is the most important glycoprotein that can be used to develop HSV vaccines and new possible therapeutic drugs. The MAbs of gD can be divided into two groups, in which one group recognizes discontinuous epitopes and another one recognizes continuous epitopes.

The gD of HSV-1 is an envelope glycoprotein that has 369 AA, and the AA sequences of gD1 of HSV-1 and gD2 of HSV-2 are 85% identical (Carfi et al., 2001). Its extracellular domain or ectodomain has 316 AA (Figure 3) (Heldwein & Krummenacher, 2008). The site of gD binding to HVEM is located at the most distal residues of N-terminal extension (residues 1 to 37) and forms a hairpin structure that can cover the whole binding site of the receptor HVEM (Carfi et al., 2001). The nectin-1 binding sites are located downstream of the first 32 residues in N-terminus of gD, as well as V34, Y38, and the cluster D215, R222, F223, which reside in the same surface of gD molecule, however, the atomic detail of nectin-1 binding to gD is still unknown (Jogger et al., 2004; Connolly et al., 2005; Spear et al., 2006; Zhou & Roizman, 2006). Most of the nectin-1 biding amino acids in gD come from the C-terminal extension, and some come from the N-terminal region (Di Giovine et al., 2011). The precise 3-O-HS-binding site is not clear yet, but the literature has reported that the binding site may overlap the HVEM binding site. The mutations in the N-terminus of HSV-1 gD can impair its binding to both HVEM and 3-O-HS, whereas has no effect on the binding to nectin-1 (Yoon & Spear, 2004). The N-terminus of gD ectodomain (1-260) contains receptors binding sites, and the 50 AA residues (260-310 residues) at the C-terminus of gD ectodomain is very important for the virus to trigger virus-cell fusion, but not for the receptor binding. The binding of its C-terminus to the N-terminus of the gD ectodomain can mask the receptor binding site (Krummenacher et al., 2005). The binding of gD with HVEM or nectin-1 requires detachment of the C-terminus from its native binding form to reveal the receptor-binding sites (Lazear et al., 2008). Then the released C-terminus induced virus-cell fusion by a cascade of reaction through gH/gL and gB.

The extracellular domain residues 1 to 23, 264 to 279, and 284 to 301 of gD are the main continuous antigenic determinants (Isola et al., 1989). The MAbs that recognize continuous epitopes in gD have a weak neutralization ability, except the MAb ID3 that can block the binding of the virus to its receptors (Figure 3) (Cairns et al., 2014).

In fact, most MAbs recognize discontinuous epitopes in gD. MAbs MC2 and MC5 have virus neutralization activity by blocking the gD–gH interaction, but not by blocking the binding of gD to receptors (Figure 3). MC2 neutralization activity can be enhanced by the non-neutralizing MAbs MC4, MC10, or MC14 mapped to the same linear epitope of AAs 262 to 272 (Figure 3). MC2 is an HSV-2 specific antibody and binds AAs 234-250 of HSV-2 gD (Figure 3). MC5 is type common and can bind the conformational epitope of gD at the residues downstream of AA 250 and those surrounding AA 77 (Figure 3). Both MC2 and MC5 neutralize HSV by interfering with the ability of gD to activate gH/gL so as to prevent from the activation of gB-induced fusion (Figure 3). gD MAbs can also be classified into groups I–VII, MAbs in groups IIa and IIc are mapped to AAs 262–272 of gD and have no neutralization activity (Cairns et al., 2014). Group I antibodies of gD are type common and can be divided into two subgroups, Ia and Ib, and the subtypes recognized absolutely distinct epitopes and can block each other (Muggeridge et al., 1988). The MAb DL11 is a member of group Ib, which can block the binding of gD to HVEM and nectin-1 (Figure 3). Part of its epitope may be located at the AA residues between 234 and 275. Group Ia MAbs can block the binding of HSV to nectin-1 but not HVEM, and the group VII MAbs can block the binding to HVEN but not nectin-1 (Krummenacher et al., 1998). The MAb DL16 is a non-neutralizing and trimer-specific antibody (Figure 3) (Bender et al., 2007). The groups VII and Ib MAbs are the only MAbs that can block the interaction of gD to HVEM. The groups Ia and II MAbs cannot block the binding of gD to HVEM but still can block the virus entry into cells through HVEM. It is suggested that virus neutralization of the two groups of MAbs occurs after the binding of gD to HVEM (Nicola et al., 1998). Sanna et al. have reported that the combination of H170 (group VII) and H128 (group Ib) can neutralize HSV-2 by blocking the binding of gD to receptors (Figure 3) better in comparison to either the MAb alone (Sanna et al., 2000). The MAb m27f has a potent neutralization activity against both HSV-1 and HSV-2, and its epitope is located at continuous AA residues 292–297 in the pro-fusion domain of gD (Figure 3). MAb m27f can abolish the viral spreading between cells (Du et al., 2017).

### 3.2.Functional domains and MAb epitopes of gH/gL

gH/gL is also a major antigen that can induce potent neutralization responses (Peng et al., 1998). The gH of HSV-1 contains 838 AAs and has a large ectodomain and a single C-terminal transmembrane anchor. gL is a protein that has 224 AAs and lacks a transmembrane region. The stable structure of the two proteins gH/gL complex can be formed at 1:1 ratio (Heldwein & Krummenacher, 2008). The gH/gL binding site of HSV-1 is located at 19–323 AAs in gH and 20-161 AAs of gL (Peng et al., 1998; Cairns et al., 2003). gL is important for the transport of gH and for gH to process and transport signal to cell surface properly as well as the folding and function of the complex (Cairns et al., 2007). The proper posttranslational processing and cell surface expression of gL is dependent on gH (Hutchinson et al., 1992). Deletion of AAs 169–224 in gL of HSV-1 can reduce the expression of gL without compromising its binding to the gH; however, the gL with the AA 161 is necessary to the expression of gH. In addition, the AAs between 155 and 161 of gL are very much vital for its chaperone-like activity and fusion function (Klyachkin et al., 2006).

Peng et al. reported that by transfecting cells with mutant gH and/or gL plasmids, binding epitopes of gH-specific and gL-specific MAbs can be mapped, and the first 323 AAs of gH and the first 168 AAs of gL can form a secreted and stable complex that can react with MAb LP11 (Peng et al., 1998). Crystal structure analysis of gH/gL complex suggests that the neutralizing MAb LP11 epitope may likely be located near residues Asp168 and Pro329, and the neutralizing MAb 52S is in the opposite face of the epitope residues Ser536 and Ala537, and the LP11 can block the binding of gH/gL to gB, and the virus-cell fusion, whereas 52S may inhibit fusion after the gB-gH-gL interaction (Chowdary et al., 2010). One study showed that the mutant virus gHΔ48/gL with an N-terminal deletion of AAs 19 to 47 of the gH2 can still bind to the liposome-like the wild-type virus (Cairns et al., 2011). The study showed that AAs 19-47 at N-terminus of gH are not necessary for gH/gL association with liposome and cell–cell fusion.

Cairns et al. have reported the characteristics of 33 MAbs of HSV-2 produced with gH_2_/gL_2_ as immunogen (Cairns et al., 2006). Among them, fourteen MAbs could bind conformation-dependent epitopes of gH_2_/gL_2_ complex and can block the virus spread, whereas the other 17 MAbs recognized linear epitopes of gH (12) or gL (5). The epitope sites of MAbs that block the fusion of HSV-2 are mostly located at the AAs 19-38 of gH and the spanning residues 182–224 of gL_2_. Atanasiu et al. reported that the epitopes of gL_2_ MAbs CHL32, CHL26 and CHL18 are separately located at AAs 146 to 165, 195 to 208, and 209 to 219 (Atanasiu et al., 2013). The AAs 168 to 178 of gL of HSV-1 is a highly antigenic and immunogenic region, and the first 323 AAs and AAs 475–648 of the gH of HSV-1 are the major antigenic sites and the second antigenic site, respectively. For gH_1_ MAbs the epitopes of H1-H11, H13, MP6-MP8, and 37S are between AAs 19 and 276 and the epitopes of H12 and 52S are between AAs 476 and 648. In addition, the epitopes of gL_1_ MAbs L1-L3, 8H4, VIII62, 82, 87, 200, and 820 are between AAs 168 and 178 (Peng et al., 1998).

### 3.3.Alignment of neutralization epitopes with functional domains of gB

The glycoprotein gB is a class III fusogenic glycoprotein, can bind to three different receptors, immunoglobulin-like type 2 receptor alpha (PILRa), non-muscle myosin heavy chain IIA (NMHC-IIA), and myelin-associated glycoprotein (MAG), and functions in virus attachment and entry (Heldwein et al., 2006; Satoh et al., 2008; Arii et al., 2010; Suenaga et al., 2010). The gB receptors are required for virus entry into cells, but the precise mechanisms on their interaction with gB are still not clear (Bender et al., 2005). The PILRa is bound by the gB AAs 53 and 480 through O-glycans (Wang et al., 2009). The glycoprotein gB consists of 904 AA residues with an amino-terminal secretory signal (AAs 1–30), an ectodomain (AAs 31–773), a transmembrane anchor (AAs 774–759), and a cytoplasmic domain (AAs 796–904) (Figure 4) (Daumer et al., 2011). gB is a conserved protein because the gBs of HSV-1 and HSV-2 can functionally substitute each other (Muggeridge, 2000).

The gB can be divided into four functional regions (FR) and four domains （I–IV) according to its MAb epitopes (Figure 4). FR1 includes the domain I (AAs 153–363) and the domain V (AAs 697–725). In the domain I there exist two internal fusion loops, FL1 (AAs 175–180) and FL2 (AAs 258–264), through which the gB can be linked to the cell membrane for cell fusion. Such linkage can be blocked by the MAbs that recognize the epitopes in the fusion loops. FR2 includes AA residues 391–410, AA residues 454–475, and a less-defined region within the domain II. MAbs against the epitopes in FR2 can block the interaction of gB with gH/gL. FR3 consists of the AA residues 500–572 in the epitopes of domain III and the AAs 573–660 of the epitopes in domain IV. MAbs against the epitopes in FR3 can block the binding of gB with cells. FR4 lies in the AA residues 31–86 in the N-terminus of gB (Bender et al., 2005; 2007; Atanasiu et al., 2010b; Stampfer et al., 2010). The membrane-proximal region (MPR) (AAs 731–773) in gB can mask the FLs so as to prevent the liposome association (Figure 4), thus it is important in modulating the association of FLs of the gB with its host cell membrane (Shelly et al., 2012).

The neutralizing MAbs of gB can be divided into several groups based on the epitopes at the four FRs. Group 1 (SS55, SS56, SS118) and group 5B (SS106 and SS144) MAbs are mapped at the FR1, whereas group 2 MAbs (H1838, H1781, and C226) at the FR2, the group 4 A MAbs (SS10, SS67, SS68, and SS69) at the FR3, and MAb H1817 at the first 12 residues of N-terminus of the FR4 (Bender et al., 2007). The AAs 600 to 690 in the ectodomain of gB is highly antigenic and contains 8 continuous epitopes and 12 discontinuous conformational epitopes (Qadri et al., 1991). Cellular entry of HSV can be effectively blocked by the antibodies to gB, such as SS55, SS120, and SS144. Such antibodies block the attachment of gB to receptors. SS55, SS120, and SS144 efficiently block the binding of gB with a liposome, however, SS106 and SS121 did not block the association of gB with a liposome, only the subdomain of functional domain 1 is involved in the liposome association (Hannah et al., 2009). MAbs SS10, SS55, and SS118 can neutralize the virus by blocking the binding of gB to the cell surface (Bender et al., 2005). The MAb C226 of gB can efficiently block the interaction of gB with gH/gL, and its epitope is conformation-dependent and, mapped to AA residues 234 to 472 (Atanasiu et al., 2010b). H1318 and H1718 recognize precise linear epitopes in the same area, which can reduce cell–cell fusion modestly. C226 can compete with H1838 for gB binding and significantly reduce cell–cell fusion (Atanasiu et al., 2010b). MAbs H1838 and H1781 recognize the peptides within the residues 390 to 410 and 454 to 473, respectively. SS106 and SS144 recognize the same epitope between the AAs 697 and 725 in gB (Heldwein et al., 2006). The gB binding of H1781 can be completely blocked by MAb C226 and such blocking is reciprocal (Bender et al., 2007). The MAb DL16 competes with H1817 for the epitopes within AAs 31–43 of FR4. The complement-dependent antibody B6 recognizes a pair of consecutive peptide spanning AAs 67 to 95 (Bender et al., 2007). In addition, Vitu et al. (2013) have reported that DL16 binds to the AAs 678-730 in FR1 of gB. *In vitro* and *in vivo*, the MAb 2c of gB recognizes discontinuous epitopes within AAs 299 to 305 and one or more additional regions of HSV-1 gB (Daumer et al., 2011). MAb 2c has potent neutralizing activity (Eis-Hubinger et al., 1993).

## 4. Conclusion and perspectives

To make better oHSVs, we reviewed the genetic engineering of the functional domains of the envelope glycoproteins to specifically target tumors and aligned the neutralization epitopes with functional domains of the respective glycoproteins for future engineering to escape host neutralization.

The gB receptors are required for virus entry into cells, but the precise mechanisms on their interaction with gB are still not clear (Bender et al., 2005). For example, the precise 3-O-HS-binding site of gB and gB induced membrane fusion may be studied in the future. Our alignment of the neutralization epitopes with functional domains of the respective glycoproteins, especially gD and gB, may be used for future engineering to escape host neutralization and avoid intratumor injection.

Systemic treatment of cancer using HSV will require targeting and antigenic stealing. We provided an in-depth review of the literature on targeting HSV and an alignment of viral neutralization sites with the functional domains of the glycoproteins involved in virus attachment and entry. First-time infection of HSV in humans would induce IgM neutralization antibody responses against HSV in about 3 weeks, whereas infected humans can maintain certain levels of neutralization antibodies as immune memory (Kampe et al., 1985). To avoid rapid neutralization on systemic delivery and the human viral neutralization immune responses, future studies would need to consider deleting as much neutralization epitopes as possible to maintain sufficient virus entry efficiency and avoid human viral neutralization responses. Our alignment of viral neutralization sites with the functional domains of the glycoproteins provided strategies to delete the neutralization epitopes when retargeting and detargeting oHSVs are designed. For example, additional deletions or modifications of the neutralization sites of the McAbs MC5, MC20, DL11, H128, MC23, LP2, HD1, and MC16 of the gDΔ6-38 of the HSV mutant R-LM113 would be worth testing to overcome neutralization and allow retargeting to cancer (Table 1 and Figure 3) (Menotti et al., 2008).

Other strategies may also be studied for oHSV to avoid immune responses and the systemic barriers to the transportation of oHSV to tumor sites through intravenous injection. oHSVs were packaged in mesenchymal stromal cells (MSCs) and progeny viruses can spread from MSCs to lung and brain metastasis tumors of breast cancer (Leoni et al., 2015). In the process of infection, the progeny of oncolytic HSV-1 is released from cells to infect adjacent cells. This is promoted by removal of the HS in the cell face through increasing the HS-degrading enzyme heparanase (HPSE) of the host cells (Hadigal et al., 2015). In addition, human antiviral NK cells preferably infect the cancer cells that are infected by oHSV, which will limit the tumor virotherapy (Alvarez-Breckenridge et al., 2012a). Valproic acid (VPA) can abrogate NK cytotoxicity activated by oHSVs, thus combination therapy of oHSVs with VPA may improve the tumor virotherapy (Alvarez-Breckenridge et al., 2012b).
